# Better force fields start with better data: A data set of cation dipeptide interactions

**DOI:** 10.1038/s41597-022-01297-3

**Published:** 2022-06-17

**Authors:** Xiaojuan Hu, Maja-Olivia Lenz-Himmer, Carsten Baldauf

**Affiliations:** Fritz-Haber-Institut der Max-Planck-Gesellschaft, Faradayweg 4-6, 14195 Berlin, Germany

**Keywords:** Data publication and archiving, Method development, Protein databases, Peptides, Density functional theory

## Abstract

We present a data set from a first-principles study of amino-methylated and acetylated (capped) dipeptides of the 20 proteinogenic amino acids – including alternative possible side chain protonation states and their interactions with selected divalent cations (Ca^2+^, Mg^2+^ and Ba^2+^). The data covers 21,909 stationary points on the respective potential-energy surfaces in a wide relative energy range of up to 4 eV (390 kJ/mol). Relevant properties of interest, like partial charges, were derived for the conformers. The motivation was to provide a solid data basis for force field parameterization and further applications like machine learning or benchmarking. In particular the process of creating all this data on the same first-principles footing, i.e. density-functional theory calculations employing the generalized gradient approximation with a van der Waals correction, makes this data suitable for first principles data-driven force field development. To make the data accessible across domain borders and to machines, we formalized the metadata in an ontology.

## Abstract (summary)

**Table:** 

Measurement(s)	Some Energy • Charge • Electrostatic Potential • Electron Density • Conformational Search
Technology Type(s)	Computational Chemistry • Computational Chemistry Statistics • Multi-Stage Sampling
Sample Characteristic - Organism	Amino Acid, Peptide, or Protein
Sample Characteristic - Environment	Gas Phase

## Background & Summary

Metal cations are essential to life: one third of the proteins in the human body require metal cofactors^1,2^. By shaping the structure of proteins, cations affect biological processes like molecular recognition or enzyme activity. Understanding the structure, dynamics, and function of metalloproteins is in the ongoing focus of many researchers, we summarize a few examples that involve simulation approaches: Tamames *et al*. analyzed zinc coordination spheres in a data set from the Protein Data Bank and complemented with DFT-B3LYP calculations^3^. Sala *et al*. investigated folding of Pyrococcus furiosus rubredoxin (PfRd), which includes an iron ion, with classical molecular dynamics (MD) simulations^4^. A calcium binding site in the blood protein von Willebrand Factor (VWF) regulates force-triggered unfolding for cleavage and therewith its activity in primary hemostasis, as illustrated by classical force-probe MD simulations^5^. Gogoi *et al*. investigated protein-metal ion binding affinities by analysing MD simulations of 49 different cation-protein complexes^6^. Metal cations can alter peptide structure by interacting with backbones and thereby enforcing non-Ramachandran geometries^7^. Cations can, by repulsion or attraction, also substantially reduce the conformational flexibility of functional sidechains^8,9^.

MD simulations of biomolecules typically rely on additive force fields, where distinct terms describe bonded and non-bonded interactions based on empirically derived parameters. Studies have shown that the accuracy of force fields is especially limited when describing interactions involving ionic species^10–13^. In particular non-bonded interactions are critical, but of course the effect that nearby located cations exert on bonds is almost impossible to grasp by the combination of bonded and non-bonded interactions in a general-purpose force field. Modeling of electrostatic interactions via pairwise Coulomb potentials is based on assigning partial charges to atoms^14^. Partial charges are derived by: (i) fitting to experimental data (GROMOS and OPLS prior 2005), e.g. by fitting partial charges to reproduce hydration free enthalpies^15,16^, (ii) deriving partial charges from QM calculations (Amber and Charmm)^17,18^, or the combination of the two strategies (OPLS after 2005)^19^.

The reliability of a force field also depends on the physics behind the formulation. The failures of established biomolecular force fields when describing cation-peptide systems may result from a central underlying assumption – modeling atoms by fixed point charges and neglecting charge transfer and polarization effects, while both are crucial to ionic systems^20–23^. Introducing more physics to the model appears a promising route to improve force fields: The inclusion of electronic polarization and charge transfer plays a central role in the next generations of biomolecular force fields^24–26^. However, including additional terms leads to force fields with way more parameters, which makes parameterization more challenging^27,28^, in particular in the absence of high-resolution experimental data of less stable conformations, i.e. higher-energy structures^29^. To summarize, we see three main challenges:

- The availability of sufficiently-accurate electronic-structure data as well as choosing the “right ways” to derive e.g. partial charges from it.
- Designing the formulation of next-generation force fields that also include, for example, charge transfer and polarization.
- Finding sets of parameters (force fields) for such potentials in the absence of experimental data at sufficient spatial and time resolution.

Thorough studies have deepened our understanding of the conformational basics of individual building blocks, e.g.^30–41^. However, these studies are highly diverse with regards to the approximations made to model and to search the potential energy surfaces (PES) of the respective molecular systems; furthermore, the data is often not available. The availability of uniform and comprehensive computational data at an appropriately accurate level of theory has the potential to substantially increase the predictive power of force fields^42^. In order to provide such amino acid data sets for force field development on consistent computational footing, we extend previous work^43^ by focusing on dipeptides as models of amino acid building blocks in polypeptide chains in complex with the divalent cations Mg^2+^, Ca^2+^, and Ba^2+^, which play prominent roles in physiology: Mg^2+^ takes structural, catalytic, and regulatory roles^44^ regulating ion channels, mitochondrial function, and cell’s pH and volume^45^. Ca^2+^ levels regulate muscle contraction, hormone secretion, metabolism, ion transport, division, *etc*^46^. Mg^2+^ and Ca^2+^ may compete for the same binding sites^47^. Ba^2+^ can cause cardiac irregularities and affect the nervous system presumably by blocking potassium channels^48^.

**Fig. 1:**
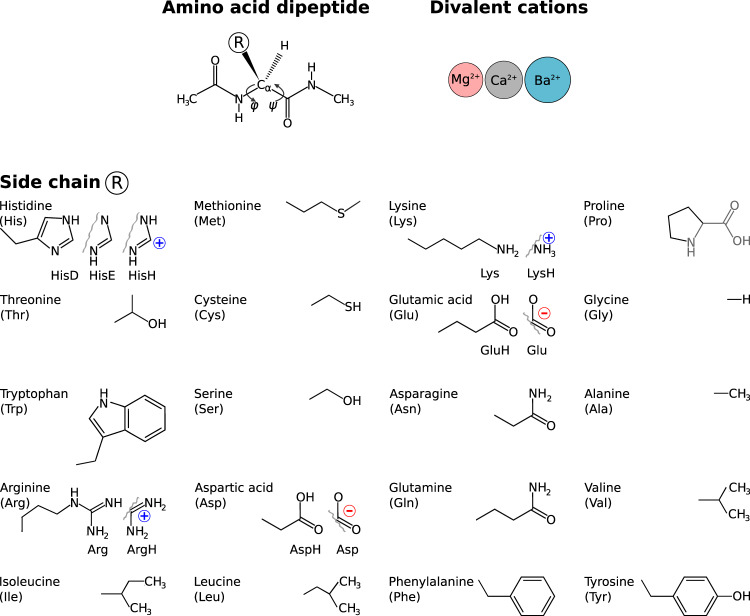
The molecular systems in this study are dipeptides of the 19 proteinogenic amino acids that differ in the side chain **R** and the proteinogenic imino acid proline. Where applicable, different protonation states were considered.

Combining these 3 cations with the proteinogenic amino acids in all meaningful side chain protonation states results in a data set that covers a wide range of molecular systems, see Fig. 1.

For the 21,909 stationary points, properties relevant to force field development were computed, details can be found in the Methods section. Making the data FAIR^49,50^ – as in findable, accessible, interoperable, and reusable – is a challenge. In particular as we want to make the data available also to experts from other domains of science or to autonomous agents. To that end, we make the data freely available and also provide ontologies. An ontology defines a common vocabulary of basic concepts in a domain and relations among them^51^. The benefit is primarily that these definitions are machine-readable. This allows for interoperability between resources and databases as well as data interpretation across data collections. Through developed ontological representation of the data set, it can be connected to upper level concepts and thereby made machine-usable, which in turn enables automatic access and querying of the data. Ultimately, researchers can share their data with experts from other domains as well as making data available to machine intelligence.

## Methods

Figure 1 summarizes the molecular systems in this study. Including the protonation states, we have to consider 26 dipeptides in 4 complexation states (bare, Ca^2+^, Mg^2+^, Ba^2+^) which results in the 104 systems for which our structure searches identified 21,909 stationary points. For each of these stationary points, not only structure and energy are provided, but also further properties relevant to force field development, namely: van der Waals energies, interaction energies as well as electron densities and derived properties like the electrostatic potential, diverse partial charge models, and effective atomic volumes. By that, our dipeptide-cation data set allows one to explicitly assess subtle, but important, effects of local changes in the electrostatic environment due to peptide-cation interaction.

### Sampling method

A hierarchical structure search that is described in detail in reference^43^ was employed to locate stationary points on the potential energy surfaces of the 104 molecular systems. The initial global conformational searches of all dipeptides with/without Ca^2+^ were performed by a basin hopping search strategy^52,53^ using the OPLS-AA force field^16^. Secondly, a refinement using density-functional theory calculations was performed. All electronic-structure calculations were performed with the all-electron, full-potential code FHI-aims utilizing numeric atom-centered basis functions^54–56^. The PBE generalized-gradient exchange-correlation functional^57^ augmented by Tkatchenko’s and Scheffler’s pairwise van der Waals correction^58^ was employed, and is referred to as PBE+vdW throughout this work. Stationary points that resulted from the FF-based pre-sampling were subjected to DFT-PBE+vdW relaxations with light settings. Next, a local first-principles based sampling step by *ab initio* replica-exchange molecular dynamics (REMD)^59,60^ employing DFT-PBE+vdW with light settings, was applied to the identified set of structures. Conformers were extracted every 10 steps from REMD trajectories and clustered with a *k*-means clustering algorithm^61^. Obtained conformers went through relaxation with PBE+vdW (light computational settings), clustering and further relaxation with PBE+vdW (tight computational settings) to obtain the final conformational hierarchies. Initial structures of Mg^2+^ and Ba^2+^ binding dipeptides were obtained by substituting Ca^2+^ cation in dipeptide binding a Ca^2+^ cation. Subsequently, those were put into the procedure from *ab initio* REMD simulations to relaxation with PBE+vdW (light computational settings) to obtain final conformers as described before. These structures were further relaxed by PBE+vdW with tight computational settings.

### Property calculations

**Fig. 2:**
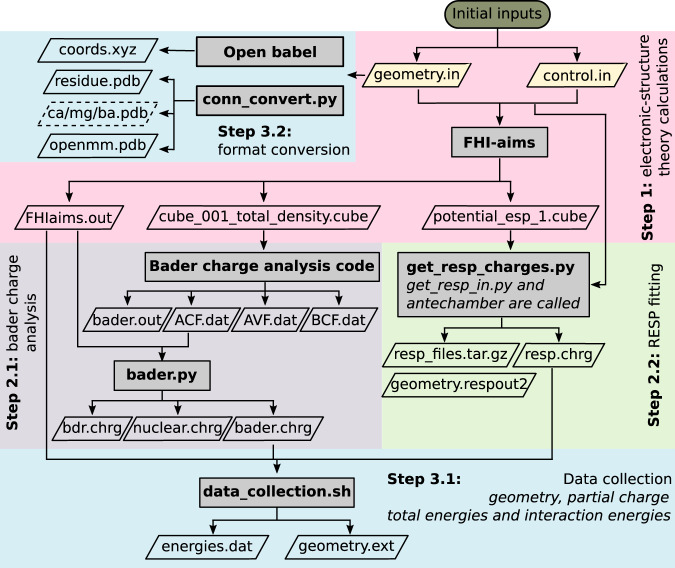
Schematic representation of the workflow employed to derive properties of each conformer. Calculation steps were displayed in boxes with different background colour. Gray boxes indicate tools employed in each step. Parallelograms represent input and output files in each step. Links to custom codes are listed in Section *Code availability*.

Property calculations were performed on all structures obtained by the sampling method described above. This includes also high energy conformers. Figure 2 shows the processes involved in the property calculations; the individual steps are described in detail below. From the PBE+vdW DFT calculations with tight computational settings using FHI-aims, we collect in **Step 1** total energies, vdW energies, interaction energies, electron densities, electrostatic potential, Hirshfeld partial charges^62^, and effective atomic volumes. Based on the effective atomic volumes *V*^ eff^ per atom we provide, the effective vdW radii (${R}_{{\rm{eff}}}^{0}$) and the polarizability (${\alpha }_{{\rm{eff}}}^{0}$) of an atom in a molecule can be calculated as follows^58,63^:

1 $${R}_{{\rm{eff}}}^{0}={R}_{{\rm{free}}}^{0}{\left(\frac{{V}^{{\rm{eff}}}}{{V}^{{\rm{free}}}}\right)}^{1/3}$$

2 $${\alpha }_{{\rm{eff}}}^{0}={\alpha }_{{\rm{free}}}^{0}\left(\frac{{V}^{{\rm{eff}}}}{{V}^{{\rm{free}}}}\right)$$

3 $$\frac{{V}_{i}^{{\rm{eff}}}}{{V}_{i}^{{\rm{free}}}}=\frac{\int {r}^{3}{\omega }_{i}(\overrightarrow{r})n(\overrightarrow{r}){d}^{3}\overrightarrow{r}}{\int {r}^{3}{n}_{i}^{{\rm{free}}}(\overrightarrow{r}){d}^{3}\overrightarrow{r}}$$

in which, ${R}_{{\rm{free}}}^{0}$ and ${\alpha }_{{\rm{free}}}^{0}$ are the vdW radii of reference free-atom and static dipole polarizability (which can be taken from either experimental data or high-level quantum chemical calculations), respectively. *V*^free^ is the volume of the free atom *in vacuo*, *r*^3^ is the cube of the distance from the nucleus of atom *i*, ${\omega }_{i}(\overrightarrow{r})$ is the Hirshfeld atomic partitioning weight for atom *i*, $n(\overrightarrow{r})$ is the total electron density, and ${n}_{i}^{{\rm{free}}}(\overrightarrow{r})$ is the electron density of the free atom *i*.

The basic property resulting from a DFT calculation is the electron density, which – for each entry in our data set – was stored on a discrete grid of points with a spacing of 0.05 Å in a rectangular volume, which spans the whole molecule plus 14 Bohr (7.4 Å) beyond the outermost nuclei. The electrostatic potential exerted by a molecule on its environment may be used to derive partial charges. To that end, for each entry in the data set, five molecular surfaces were created by increasing the van der Waals radii of all atoms in the molecule (molecule with cation) by factors between 1.4 and 2.0. Points on these surfaces were represented in a cubic grid of each 35 grid points in *x*, *y*, and *z* direction. For these points, the electrostatic potential was evaluated. For biomolecular force fields, atomic partial charges are a crucial ingredient for computing the pairwise Coulomb term of the non-bonded interactions. We provide three types of partial charges:

- Hirshfeld atomic charges, computed by FHI-aims, were derived based on the Hirshfeld partitioning scheme^58,62^. The Hirshfeld atomic charge *q*_*i*_ of atom *i* is given by

4 $${q}_{i}={Z}_{i}-\int {n}_{i}(\overrightarrow{r}){d}^{3}\overrightarrow{r}$$

where *Z*_*i*_ refers to the corresponding atomic number, and ${n}_{i}(\overrightarrow{r})$ is the associated electron density associated with atom *i*.

5 $${n}_{i}(\overrightarrow{r})={\omega }_{i}(\overrightarrow{r})n(\overrightarrow{r})$$

where $n(\overrightarrow{r})$ denotes the total electron density, ${\omega }_{i}(\overrightarrow{r})$ is the Hirshfeld atomic partitioning weight for atom *i*. ${\omega }_{i}(\overrightarrow{r})$ is given by

6 $${\omega }_{i}(\overrightarrow{r})=\frac{{n}_{i}^{{\rm{free}}}(\overrightarrow{r})}{{\sum }_{A}^{Allatoms}{n}_{A}^{{\rm{free}}}(\overrightarrow{r})}$$

- Bader charges were being computed in **Step 2.1** using the Bader Charge Analysis tools^64–66^ provided by the Henkelman group based on the electron density cube file produced in Step 1. The atoms in molecules (AIM) partitioning method uses what is called zero flux surfaces to distribute electron density among the atoms. Such zero flux surface is a two-dimensional surface on which the charge density is a minimum perpendicular to the surface. In molecular systems, the charge density typically reaches a minimum somewhere between pairs of neighboring nuclei. This can be seen as the natural place to separate atoms from each other. These borders between atoms define the electron density region associated with a given atom, from which the partial charges are being calculated.
- In **Step 2.2**, RESP partial charges^67–69^ were computed using Antechamber^70^ from the AmberTools package^71^. A two-stage restrained electrostatic potential (RESP) fitting procedure^67^ was employed as implemented in Antechamber.

In the final **Steps 3.1 and 3.2**, data was collected and files converted to established formats. Geometry information is provided in three formats: the FHI-aims input format, the xyz format generated by Open Babel^72^, and PDB files that are readable by the CHARMM-GUI portal^73^ and the openMM7 package^74^. Connectivity and atom type information – needed for the PDB format – was gathered based on atomic distances by the Python script conn_convert.py. Furthermore, energies and partial charges were tabulated for convenient usage. Interaction energies E_inter_ between cation and dipeptide were calculated as follows:

7 $${E}_{{\rm{inter}}}={E}_{{\rm{complex}}}-{E}_{{\rm{dipeptide}}}-{E}_{{\rm{cation}}}$$

where *E*_complex_ corresponds to the potential energy of the dipeptide-cation complex, *E*_dipeptide_ is the potential energy of the dipeptide alone fixed in the cation bound conformation, and *E*_cation_ is the potential energy of the isolated cation.

Further data and properties can be extracted from the raw and normalized data^75^ that is available from the NOMAD Repository and Archive^76^. The data set was deposited as populated ontology in OWL format^77^ in the EDMOND repository of the Max Planck Society. The construction of the ontology is described in the following subsection.

### Ontology construction

Ontology construction is an iterative process involving many steps from defining common vocabularies, identifying the most important concepts and their relations to modelling such concepts in a semantically correct and still useful and applicable way. It can be used to enrich, annotate, and link data that is then called *linked data* and usually expressed in a semantic triple format consisting of *subject*, *predicate*, and *object*^78^. The main components of an ontology are classes, properties, individuals and axioms. Classes are the focus of most ontologies and are descriptions of concepts in a domain and represent a specific set of individuals. “Ala” is a class in the Amino Acid domain, thus each single Ala conformer in our data set is an individual of class “Ala”. Properties describe features and attributes of classes and individuals. Properties can connect classes and individuals. For example, *hasProperty* can connect classes “Ala” and “Charge” as a property. Axioms are statements that all together define what is the truth in a given domain. In this work, the ontology builder Protégé^79^ and the python package Owlready2^80^ were employed to build ontologies in the OWL2 Web Ontology Language (http://www.w3.org/TR/owl2-overview) which is based on RDF – the Resource Description Framework (http://www.w3.org/TR/rdf-primer). Subjects and predicates are named using Internationalized Resource Identifiers (IRIs) (https://tools.ietf.org/html/rfc3987), while the object position can be filled by an IRI or a literal value (e.g. string or number). Ontologies created in this work have been tested with the OWL reasoner FACT++^81^.

## Data Records

Raw data and normalized data of the DFT calculations for this amino acid dipeptide data set is available from the NOMAD repository (http://nomad-repository.eu) via the 10.17172/NOMAD/2021.02.10-1^75^. The NOMAD Archive contains all raw input, output, and property calculation files for download, while the NOMAD Repository contains normalized data, i.e. a digest of the DFT calculations. Data in the NOMAD Repository and Archive is provided on the basis of the Creative Commons Attribution 3.0 License (CC BY 3.0) as it is stated in the NOMAD terms (https://nomad-lab.eu/terms).

The extracted data in form of a populated ontology in OWL format is available download via the 10.17617/3.5q10.17617/3.5q^77^ under the Creative Commons Attribution 4.0 license (CC BY 4.0). In the following two subsections, we briefly introduce the data and the concept of the provided ontology.

### DFT data set

**Fig. 3:**
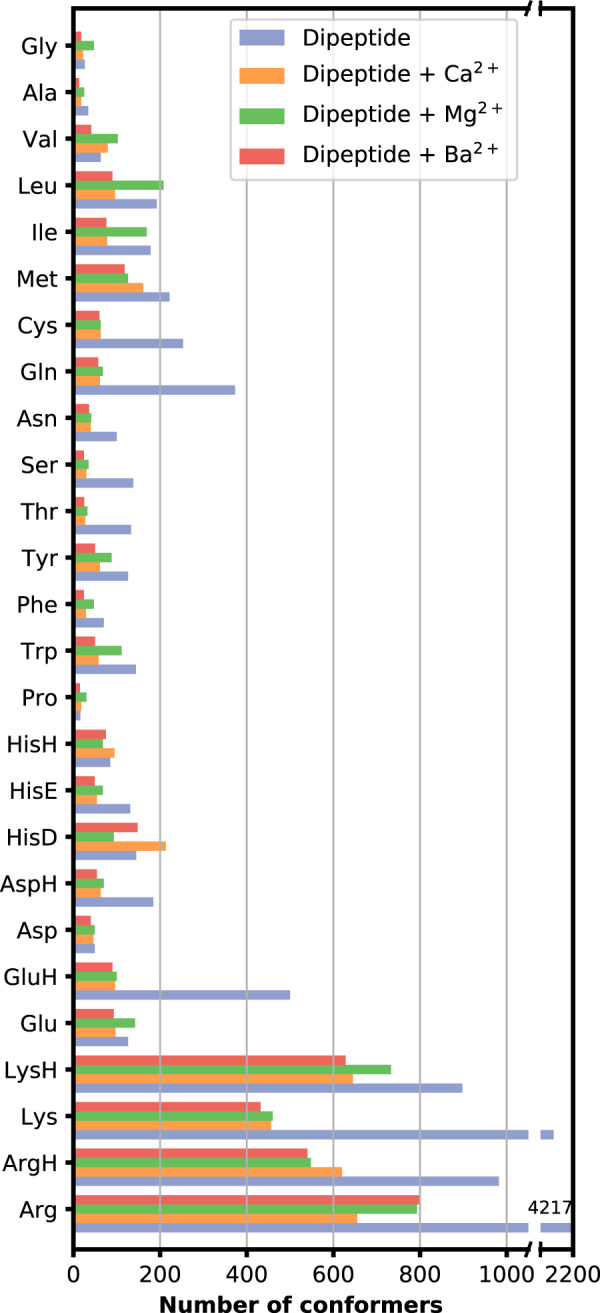
Numbers of stationary points of each molecular system covered in this study.

The distribution of the 21,909 stationary points of the amino acid dipeptide (plus cation) systems over the different amino acid building blocks is summarized in Fig. 3. This data is in particular intended for training energy functions in machine learning approaches in the context of force field development and parameterization. Consequently, it consists not only of geometries with total energies for preferred low-energy conformers. Instead, DFT-PBE+vdW calculations also included high-energy conformers. The data we provide is particularly focused on parameterizing non-bonded interactions: The above-mentioned cation-peptide interaction energies were already used to tune force fields parameters of non-bonded interactions^26,82^. The comparison to DFT-based vdW energies computed with the Tkatchenko-Scheffler formalism^58^ is useful to evaluate or adjust the non-bonded Lennard-Jones parameters *ε* and *σ*. Importantly, due to the spread over high and low energy conformations, diverse substructures and environments (due to cation binding), a range of partial charge values is sampled that informs about polarization and charge transfer. To that end, the electronic structure is simplified into partial charge models, based on Hirshfeld partitioning or Bader AIM analysis of the electron density. The electron density, in combination with the nuclear charges, also defines the electrostatic potential (ESP) around the molecule, which can be used to derive force field parameters related to electrostatic interaction^83^. The electron density has been used before to derive environment-specific force fields^84^. Electron densities for a large set of molecules have been used to predict partial charges based on machine learning^85,86^, to that end, an average over similar substructures in different molecules was used.

**Fig. 4:**
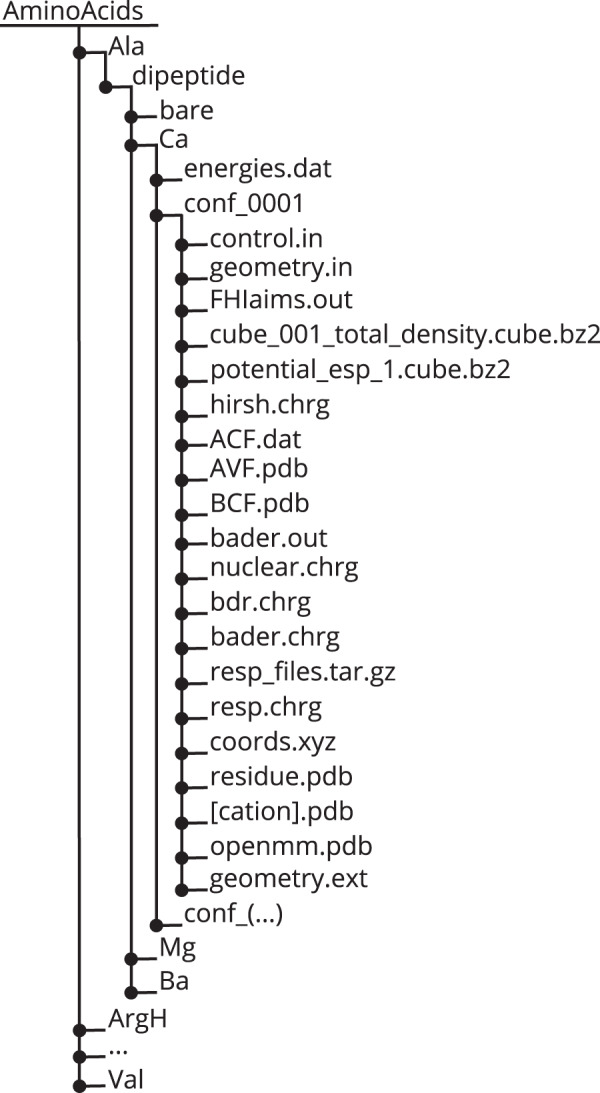
Schematic representation of the folder structure of the data. Each folder, as exemplified for the Ca^2+^-coordinated cysteine dipeptide, contains multiple properties per system.

The data is first of all made available as a set of files. The different files, their content, and which programs to read or write them are given in Table 1. A direct way to access the data is to download the compressed archive^75^ and browse the folder structure that is given in Fig. 4 or download from the same source the normalized data in json-files.

**Table 1:** List and description of file types in the data set.

File name	Description	Code/Format
**FHI-aims Input Files**		
geometry.in	Cartesian coordinates of the complexes	FHI-aims
control.in	Input file with technical parameters for electronic structure calculations	FHI-aims
**FHI-aims Output Files**		
FHIaims.out	Main output the electronic structure calculations, contains: total energy, vdW energy and effective atomic volume etc.	FHI-aims
cube_001_total_density.cube.bz2	Cube file representation of the electron density (bzip2 compressed)	FHI-aims
potential_esp_1.cube.bz2	Cube file representation of the electrostatic potential (bzip2 compressed)	FHI-aims
hirsh.chrg	Hirshfeld charges	Self-made
**Geometries**		
coords.xyz	Coordinate file	*xyz* format
residue.pdb	Coordinate file	CHARMM
[cation].pdb	Separate coordinate file for each of the cations Ca, Ba, Mg	CHARMM
openmm.pdb	Coordinate file	OpenMM
**Bader AIM calculations**		
ACF.dat, AVF.dat, BCF.dat, bader.out	Information of Bader charge analysis	Bader
nuclear.chrg, bdr.chrg	Information of Bader charge analysis	Self-made
bader.chrg	Bader charges	Self-made
**RESP calculations**		
geometry.respout2, resp_files.tar.gz	RESP charge information	Antechamber
resp.chrg	RESP charges	Self-made
**Aggregated output**		
geometry.ext	Collection of coordinate and charge information	Self-made
energies.dat	Collection of total energy and interaction energy	Self-made

This way of representing data however limits the automated access to the data by artificial agents or by researchers from other domain, as the metadata to the data is somewhat hidden. In order to alleviate this, the next section details the ontology which we developed in order to provide an extensible, machine-interpretable and machine-usable model for the automated access and post-processing of the data set.

### Ontology

**Fig. 5:**
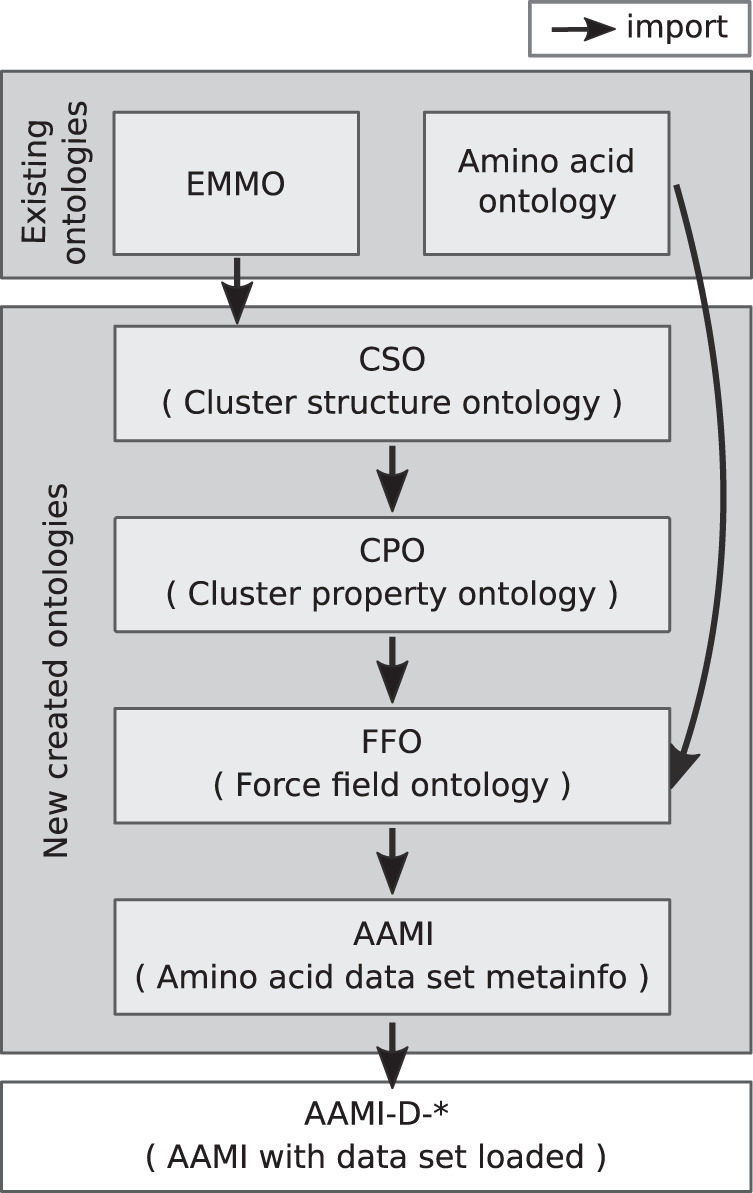
Hierarchy of the ontologies linked to amino acid-cation meta-info (AAMI). Details of the ontologies and relations among them are described in Section *Ontology*.

AAMI (Amino Acid Meta-Info) is an ontology created “bottom-up” to specifically represent the meta-information of this amino acid-cation data set in a machine-understandable and machine-processable way. AAMI does not only contain metadata of properties, it also covers processes of analysis, such as inputs, outputs, and tools in each process and their roles, which further makes data interpretable and understandable. Two existing ontologies were re-used in AAMI: the European Materials Modelling Ontology (EMMO) (https://emmc.info/emmo-info), which provides a representational framework for materials modelling and characterization knowledge, and the Amino Acid Ontology (http://bioportal.bioontology.org/ontologies/AMINO-ACID), which provides structured knowledge of amino acids and their properties. By reusing existing terms in EMMO and Amino Acid Ontology rather than creating the ontology from scratch, terms in AAMI were connected to upper level concepts and can be potentially linked to further ontologies. Moreover, users are able to take advantage of data and annotations that are already used in those ontologies and can by that also rely on concepts that were already agreed upon in a bigger community. The primary aim of AAMI is to make our data set FAIR (Findable, Accessible, Interoperable, and Reusable)^49^, in particular accessible, interoperable and reusable. The elements of AAMI can be found in Fig. 5. In the AAMI ecosystem, we created:

1. The cluster structure ontology (CSO) represents concepts and relations for structure description of non-periodic systems, EMMO was imported, and 351 classes and 2053 axioms were created.
2. The cluster property ontology (CPO) describes properties of non-periodic systems. CSO was imported, and 450 classes and 2984 axioms were created.
3. The force field ontology (FFO) represents concepts in force fields, e.g. atom type and atom class. Amino acid ontology and CPO were imported, and 563 classes and 4453 axioms were created.
4. AAMI represents concepts and relations in the amino acids-cation data set. FFO was imported, and 787 classes and 5466 axioms were created.
5. The different instances of AAMI-D-* are knowledge graphs created from the data set in this study. Such graph is build by populating AAMI with the data for an amino acid, e.g. ALA, ARG, *etc*., from this data set. The asterisk represents the name of the corresponding amino acid.

**Fig. 6:**
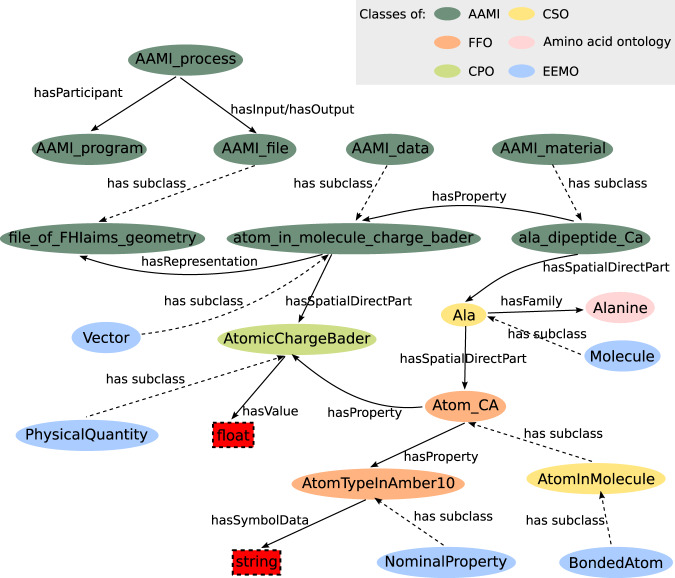
Partial high-level class structure of AAMI ontology. Ovals represent classes, where classes from different ontologies are color coded. Rectangles represent literals. Solid lines are properties and dotted lines represent the relation of ‘*has subclass*’.

Partial high level class organization and some of the classes and relations of AAMI are shown in Fig. 6 to give an overview of the organization of the ontology and how terms from each ontology are related to each other.

The primary use of AAMI is to annotate database records. However, since ontologies were developed with the OWL2 Web Ontology Language, which represents data by sets of subject-predicate-object statements, so-called *triples*, the underlying computational logic enables automatic inference and querying over data repositories. In principle, any question framed in the respective mathematical logic can be answered in a finite number of steps. However, such reasoning capabilities are currently limited to description logic. Data query can be done with the ontology and linked data query language, SPARQL (https://www.w3.org/TR/sparql11-query). A user can query for sub-classes, relations between classes, functional annotation, and so on. Stardog Studio (https://www.stardog.com/studio) can be used as a *triple store* and employed to perform the SPARQL queries. A tutorial of SPARQL query language using Stardog Studio can be found in the following link: https://www.stardog.com/tutorials/sparql/. We provide two sample queries in this work to guide users to build their own queries.

Before any queries, a set of namespace prefixes were declared to abbreviate IRIs, e.g. the knowledge graph of alanine dipeptide was imported as an example under the PREFIX ala.

The main query form in SPARQL is a SELECT query. A SELECT query has two main components: a list of selected variables and a WHERE clause for specifying the graph patterns to match. For example, according to the graph shown in Fig. 6, we can query for Bader charges of atoms which have atom type of “1” in Amber10 with a SELECT query as follows:

The resulting list shows all atoms of type “1” in Amber10, *i.e*. hydrogen atoms bound to a peptide bond nitrogen, and their Bader charges:

Another useful query is DESCRIBE, which returns all the outgoing edges of a node. DESCRIBE is most useful when we don’t know much about the ontology and want to quickly see the terms used in the triples. For example, we can query “describe individuals which belong to class Atom_C” with DESCRIBE query within the alanine dipeptide knowledge graph:

In the following, we display part of the output of the query, from which we can see that an individual “Atom_C_9_alaD_Ca_conf_0017” belongs to class “Atom_C” and has properties of “AtomicChargeBader_1.35427”, “position9” and so on.

With tools like Stardog Studio, the results of such query can be written out in various file formats for further usage, e.g. XML,JSON-LD for triples output or CSV for tabular output.

## Technical Validation

The reliability of the DFT-PBE + vdW level of theory for amino acids and amino acids binding divalent cations was evaluated before^43^. In this reference, single-point energy calculations were performed on all structures of alanine (Ala) and phenylalanine (Phe) amino acids in isolation, as well as binding with a Ca^2+^ cation employing Møller-Plesset second-order perturbation theory (MP2)^87,88^. For the structures of the amino acids Ala and Phe without cation bound, mean absolute errors (MAE) within chemical accuracy (1 kcal/mol) were estimated for PBE + vdW. A different long-range dispersion method, the many-body dispersion model (PBE + MBD)^89^, didn’t show significant improvements for isolated amino acids. Also the usage of a hybrid exchange-correlation functional, PBE0 (PBE0 + MBD)^89^, did not significantly improve the MAEs. However, the maximum error of Phe was reduced from 2 kcal/mol to 1.3 kcal/mol. MAEs were slightly higher with PBE + vdW when Ca^2+^ was involved. They reached 1 kcal/mol and 2 kcal/mol for Ala + Ca^2+^ and Phe + Ca^2+^, respectively. Employing both, many-body dispersion and the hybrid functional PBE0, improved the MAE to about 1 kcal/mol. In a manuscript on histidine-zinc interactions^11^, DLPNO-CCSD(T)^90,91^ was employed to benchmark several DFAs as well as the wave function-based MP2 method. The evaluated systems are (a) negatively charged acetylhistidine (AcH) with and without a Zn^2+^ cation, and (b) neutral AcH with and without a Zn^2+^ cation. The results showed that PBE+vdW gave an acceptable accuracy. In conclusion, PBE+vdW appears to be a valid starting point for studies on cation-peptide systems.

The validation of the sampling method can be elucidated by the work in ref. ^92^. A genetic algorithm was employed to do the sampling of the low-energy segment in the conformational space of seven dipeptides: Glycine (Gly), Alanine (Ala), Phenylalanine (Phe), Valine (Val), Tryptophan (Trp), Leucine (Leu), Isoleucine (Ile). Conformers from our previous data set^43^ were used as reference points and both studies agree in their overall structure findings.

The potential usage of our data set has been confirmed in ref. ^26^. In this work, our data set was used to assess the accuracy of existing FFs by their abilities to reproduce quantum mechanical (QM) interaction energies of Ca^2+^-dipeptide. By relating the parameter space to conformational space, the utility of our data set as a reference for future optimization of polarizable force fields is illustrated.

**Fig. 7:**
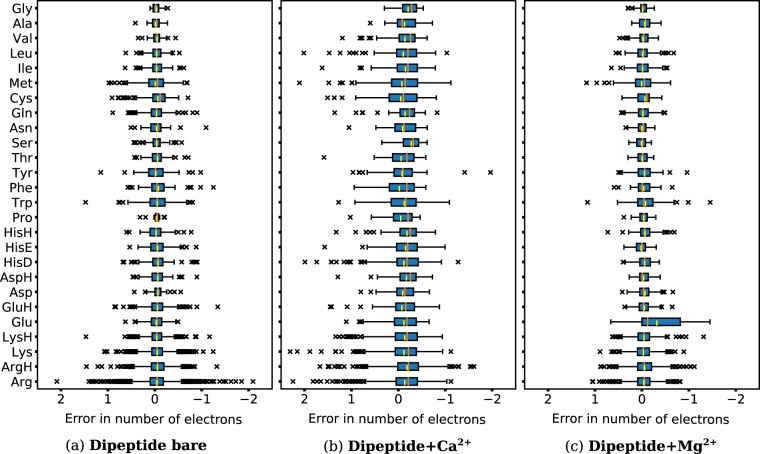
Error in numbers of electrons from Bader analysis of Dipeptide (**a**) bare, (**b**) with Ca^2+^ and (**c**) with Mg^2+^. The upper and lower lines of the rectangles mark the 75% and 25% percentiles of the distribution, the orange and yellow horizontal lines in the box indicate the median (50% percentile) and mean value, and the upper and lower lines of the “error bars” depict the 99% and 1% percentiles. Crosses represent the outliers.

An assessment of the reliability of Bader charge analysis of bare dipeptides as well as dipeptide-Ca^2+^ and dipeptide-Mg^2+^ complexes is shown in Fig. 7. The number of electrons from Bader charge analysis yielded high errors in some structures of dipeptide-Ca^2+^, reaching 2 electrons. This error apparently results from too wide grid spacing at regions of rapid density change (near “heavy” cores) when writing the electron density to cube files, the input for the Bader analysis code. Changes in electron density are particularly large close to the cations in the investigated clusters, so in principle grid spacings adjusted to the respective systems would be required. Overall, however, the mean errors of each amino acid are around 0. The errors of dipeptide-Mg^2+^ have the same trend, but are smaller than the errors of dipeptide-Ca^2+^ due to the smaller radius of Mg^2+^. Ba^2+^ is much heavier than Ca^2+^ and Mg^2+^, the rise in density close to the atomic center is much steeper. To analyze the Bader charges of dipeptide-Ba^2+^ complexes, a much smaller grid spacing is needed. However, this will result in electron density cube files that are impractically large for an overview study of this extend. So in this work, we did not present the electron density and Bader charges of dipeptide-Ba^2+^ complexes.

## Usage Notes

Attention, the download of the whole archive of raw data is about 1.5 TB in size (compressed). Structures in this data set are stationary-point geometries, most of them can be expected to be minima, yet there are certainly also saddle points. All files in the NOMAD repository can be downloaded through curl based on upload and entry IDs (variables: upload_id and entry_id below). The command below downloads all files in one calculation:

The metadata for the DFT calculations can in part be browsed at the NOMAD Archive page (https://www.nomad-coe.eu/the-project/nomad-archive/archive-meta-info). There are numerous tools to perform SPARQL queries, e.g. Stardog Studio (https://www.stardog.com/studio), Protégé^79^, RDFLib (https://github.com/RDFLib/rdflib), Apache Jena (https://jena.apache.org), and so on. The licenses of Protégé, RDFLib, and Apache Jena are BSD 2-Clause, BSD 3-Clause and Apache License 2.0, respectively; using Stardog Studio requires for a license from the developers.

## Data Availability

All custom codes used in this study have been uploaded to Github^93^.
